# Gene therapy for monogenic liver diseases: clinical successes, current challenges and future prospects

**DOI:** 10.1007/s10545-017-0053-3

**Published:** 2017-05-31

**Authors:** Julien Baruteau, Simon N. Waddington, Ian E. Alexander, Paul Gissen

**Affiliations:** 1 Genetics and Genomic Medicine Programme, Great Ormond Street Institute of Child Health, University College London, London, UK; 2 Metabolic Medicine Department, Great Ormond Street Hospital for Children NHS Foundation Trust, London, UK; 3 Gene Transfer Technology Group, Institute for Women’s Health, University College London, London, UK; 4 Wits/SAMRC Antiviral Gene Therapy Research Unit, Faculty of Health Sciences, University of the Witwatersrand, Johannesburg, South Africa; 5 Gene Therapy Research Unit, The Children’s Hospital at Westmead and Children’s Medical Research Institute, Westmead, Australia; 6 Discipline of Child and Adolescent Health, University of Sydney, Sydney, Australia; 7 MRC Laboratory for Molecular Cell Biology, University College London, London, UK

## Abstract

Over the last decade, pioneering liver-directed gene therapy trials for haemophilia B have achieved sustained clinical improvement after a single systemic injection of adeno-associated virus (AAV) derived vectors encoding the human factor IX cDNA. These trials demonstrate the potential of AAV technology to provide long-lasting clinical benefit in the treatment of monogenic liver disorders. Indeed, with more than ten ongoing or planned clinical trials for haemophilia A and B and dozens of trials planned for other inherited genetic/metabolic liver diseases, clinical translation is expanding rapidly. Gene therapy is likely to become an option for routine care of a subset of severe inherited genetic/metabolic liver diseases in the relatively near term. In this review, we aim to summarise the milestones in the development of gene therapy, present the different vector tools and their clinical applications for liver-directed gene therapy. AAV-derived vectors are emerging as the leading candidates for clinical translation of gene delivery to the liver. Therefore, we focus on clinical applications of AAV vectors in providing the most recent update on clinical outcomes of completed and ongoing gene therapy trials and comment on the current challenges that the field is facing for large-scale clinical translation. There is clearly an urgent need for more efficient therapies in many severe monogenic liver disorders, which will require careful risk-benefit analysis for each indication, especially in paediatrics.

## Introduction

The liver is a key-regulator of multiple complex metabolic pathways and the hepatocyte is a primary cell type affected in numerous inherited genetic/metabolic diseases (Clayton 2002). Despite a wide range of disease-specific conventional therapies, liver replacement therapies remain a valid strategy and even a potential cure for many monogenic liver disorders due to the ability to restore the defective pathway (Sokal 2006). Liver replacement options include whole or partial organ (Spada et al 2009), or hepatocytes transplantation (Dhawan et al 2006). The shortage of donors, the associated mortality/morbidity and need for immunosuppression, however, often limit this option to severely affected patients and those aged more than 3 months or weighing greater than 5 kg (Haberle et al 2012). In the past decade, liver-directed gene therapy has emerged as a promising alternative to transplantation in monogenic liver disorders.

## Overview of gene therapy development: reaching maturity

Gene therapy, by providing additional functional gene copies, has been considered for decades as an attractive option for treatment of monogenic disorders (Wirth et al 2013). According to the Gartner hype cycle, a graphical representation depicting the maturity of novel technologies, gene therapy reached its “peak of inflated expectation” in the mid-1990s which was paralleled by a rapid rise in clinical trial activity and the publication of early proof-of-concept studies for genetic and acquired conditions such as adenosine-deaminase deficiency (ADA-SCID) (Blaese et al 1995; Bordignon et al 1995) and brain tumours, respectively (Puumalainen et al 1998). This period of inflated expectation was critiqued in the Orkin-Motulsky report commissioned by the National Institute of Health (Orkin and Motulsky 1995). While acknowledging the extraordinary promise of gene therapy, the report emphasised the need for greater focus on gene transfer technology and the basic science of gene transfer. Soon after, the field plunged into its “trough of disillusionment” following the death of a young adult, Jesse Gelsinger, in a clinical trial for ornithine transcarbamylase (OTC) deficiency (Raper et al 2003). Optimism arising from the subsequent clinical success in the treatment of X-linked severe combined immunodeficiency (SCID-X1) (Cavazzana-Calvo et al 2000) was soon dampened by the occurrence of leukaemia in five out of 20 patients secondary to insertional mutagenesis (Hacein-Bey-Abina et al, 2003a, b; Fischer et al 2010; Mukherjee and Thrasher 2013), causing the death of one participant (Mukherjee and Thrasher 2013). Resultant concerns over gene therapy were further compounded by growing awareness of the challenges imposed by vector-induced immune responses (Mingozzi and High 2007). Disbelief and doubt followed, leading to a decline in financial investment (Ledley et al 2014). In parallel, these adverse events motivated researchers to seek a better understanding of the challenges posed by disease pathophysiology and to develop safer and more efficient vectors. Recent clinical successes in various inherited orphan diseases such as Leber’s congenital amaurosis (Bainbridge et al 2008; Cideciyan et al 2008; Maguire et al 2008), X-linked adrenoleukodystrophy (Cartier et al 2009), metachromatic leukodystrophy (Biffi et al 2013) and haemophilia B (Nathwani et al 2011) and the first market authorisation granted by the European Medicines Agency (EMA) in 2012, to Glybera® for lipoprotein lipase deficiency (Bryant et al 2013), are driving the field up the “slope of enlightenment” and onto the “plateau of productivity”. As a result of this success, various biotechnology companies dedicated to gene therapy development have been created and received substantial financial investment (Cassiday 2014). In parallel, the number of gene therapy-based clinical trials has risen rapidly in recent years (http://www.abedia.com/wiley/(accessed 2017 Jan 06); Ginn et al 2013).

## Strategies for hepatocyte-directed gene transfer

**Fig. 1:**
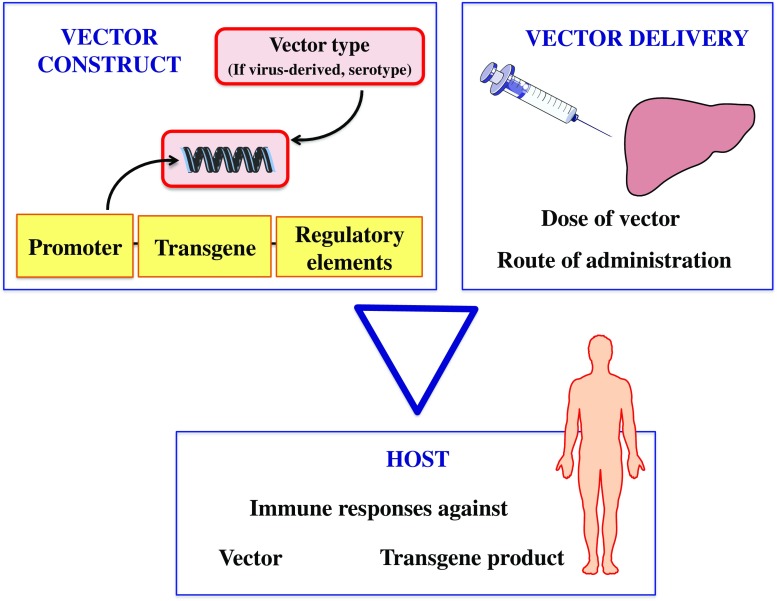
The triad to consider for successful gene therapy

A growing toolbox is available for gene transfer, which has been the preferred approach in recent human trials targeting hepatocytes. Various elements in the choice of transgene expression cassette design, mode of delivery and the subset of patients targeted influence the efficacy of gene therapy (Fig. 1).

### Parameters of vector delivery

The mode of transgene delivery is crucial: i) local injection allows highly selective expression, but in a limited area. Conversely an intravenous injection allows a broad distribution balanced by non-specificity. Injections in the hepatic artery or the portal vein improve the selectivity but require cannulation with its associated risks (Fumoto et al 2013). Peripheral intravenous delivery provides similar transduction compared to intrahepatic or intraportal routes for AAV vectors (Sarkar et al 2006; Nathwani et al 2007); ii) higher doses of vector achieve greater transduction, but may generate more severe immune responses (Raper et al 2003; Mingozzi and High 2013).

### Host pre-sensitisation or acquired immune responses

The immune response against the vector and/or the transgene product might preclude the expected therapeutic effect (Jooss and Chirmule 2003; Zaiss and Muruve 2008; Wold and Toth 2013).

Immune memory of pre-exposure to wild-type viruses can prevent efficient hepatocyte transduction by pre-existing neutralising antibodies and might account for differences in the severity of immune responses observed after systemic injection. Pre-immunisation against the transgene product can occur when the recombinant transgenic protein has been administered. For example, in haemophilia B patients, this can result in the generation of anti-factor IX antibodies when treated by recombinant factor IX (Armstrong et al 2014). Accordingly prior immunisation needs to be carefully considered in clinical trial enrolment criteria.

Acquired immune responses after systemic gene delivery are common. Innate immune responses are triggered by antigen presenting cells such as dendritic cells or macrophages initiating the release of proinflammatory cytokines (interleukins 1 and 6, tumour necrosis factor α (TNFα), type I interferon α and β) via stimulation of Toll-like receptors (TLR). Specific and long-lasting antigen-specific immune responses are mediated by B- and T-cells and involve secretion of neutralising antibodies and CD8^+^ cytotoxic T lymphocytes, respectively, regulated by the recruitment of helper and regulatory CD4^+^ T cells. Whatever the viral vector considered, immune responses share various similarities, and must always be carefully considered (Bessis et al 2004; Tang et al 2006; Annoni et al 2013; Calcedo and Wilson 2013; Basner-Tschakarjan and Mingozzi 2014).

### Design and selection of the gene transfer vector

The *transgene expression cassette* contains i) a transgene, which is commonly a cDNA and may be codon-optimised to achieve higher expression of the transgene product, ii) an enhancer/promoter, the selection of which determines the level of transgene expression, cell-type restricted specificity of expression and also influences the risk of insertional mutagenesis, iii) various pre- and/or post-regulatory elements to stabilise transgene mRNA and therefore increase the yield of transgene product, e.g. addition of an intron downstream of the promoter containing a bacterial replication origin (Lu et al 2017) or the woodchuck hepatitis virus post-transcriptional regulatory element (WPRE) (Lipshutz et al 2003), respectively.

## Several options are available to deliver the *transgene expression cassette* to the target cell/organ

Injection of *naked DNA,* either as *plasmids* (Doenecke et al 2010; Oishi et al 2016) or *mini-circles* (Viecelli et al 2014; Hou et al 2016; Wu et al 2016), is a simple mode of transgene delivery which lends itself to local delivery and is relatively non-immunogenic compared to some viral vector approaches (Wolff and Budker 2005). Mini-circles are devoid of plasmid backbone DNA; this may enhance transgene expression by overcoming heterochromatin formation and avoiding inflammation triggered by bacterial DNA (Mayrhofer et al 2009). These approaches allow easy production of therapeutic material with a good safety profile and capable of eliciting long-lasting transgene expression in post-mitotic tissues (Wolff and Budker 2005; Kay et al 2010). These approaches have been employed in ∼17% of gene therapy trials so far (http://www.abedia.com/wiley/(accessed 2017 Jan 06)). The hydrodynamic injection technique, developed in small animal models, consists of injecting DNA plasmids or mini-circles in a large vehicle volume to flood the liver with pressurised DNA solution; this disrupts vascular endothelium, and allows high levels of transgene expression in small or large animal models (Liu et al 1999). Although hydrodynamic injections are difficult to translate to humans, intravascular hydrodynamic procedures with partial catheterisation for liver-directed gene delivery have shown some success in large animals (Sendra et al 2016; Yokoo et al 2016). However, current translatable options of non-viral approaches remain limited. Therefore viral vectors, acting as Trojan horses to increase transduction efficiency, are frequently considered.

*Non-viral vectors* are synthetically produced biological particles, in which the transgene is encapsulated, or complexed, and released at the target site. Various engineered nanoparticles exist, e.g. liposomes or/and polymers (Chira et al 2015). These options have several advantages: easy production, no restriction of the transgene size, and a reliable safety profile. Limitations are the stability of these particles, cellular uptake and a limited ability to achieve long-lasting transgene expression (Elsabahy et al 2011). These are therefore suboptimal delivery vehicles for liver-directed clinical trials.

*Virus-derived vectors* represent an attractive approach based on their relatively efficient transduction human cells. The main vectors that have been used in clinical trials are derived from adenoviruses (21%), retroviruses (excluding lentiviruses) (19%), adeno-associated viruses (7%) and lentiviruses (6%) (http://www.abedia.com/wiley/(accessed 2017 Jan 06)).

## Retroviral vectors

*Gamma-retroviruses* such as murine leukaemia viruses (MLVs) are RNA viruses encoding *gag*, *pol* and *env* genes flanked by long terminal repeats (LTRs), which carry enhancers/promoter elements and are required for integration. After transduction of the target cell, reverse-transcription generates a double-stranded DNA copy of the proviral genome, which then integrates in the host genome providing long-term transgene expression. Gamma-retroviruses are unable to transduce non-dividing cells as the nuclear membrane prevents retroviral vectors from entering the nucleus (Miller et al 1990). This explains why this vector is more often considered for ex vivo gene therapy in which cultured target cells are stimulated to replicate and then transduce. *Lentiviruses* are a class of retroviruses, the most widely known of which is HIV1. Lentiviral vectors are able to transduce dividing and non-dividing cells, which broadens their application. Retroviral vectors have relatively large transgene capacities (7.5 kilobases (kb)) (Verma and Somia 1997). In fact payloads exceeding 14 kilobases have been packaged into lentiviral vectors (Counsell et al, 2017).

*Clinical applications and limitations:* In an early gene therapy trial, a γ-retroviral vector was used in an ex vivo approach with autologous hepatocytes in five patients with homozygous familial hypercholesterolemia. This showed a mild improvement of lipid profiles in two patients with a very low rate of stable engraftment at 4 months after gene therapy (Grossman et al 1994; Grossman et al 1995). Several limitations emerged from this trial: i) the need for two invasive procedures, i.e. the surgical resection of a liver lobe to obtain sufficient primary hepatocytes for transduction as hepatocytes cannot be expanded in culture and reinjection of transduced hepatocytes into the portal circulation via a local catheter, with the associated risks of venous thrombosis, catheter misplacement and haemorrhage (Grossman et al 1994; Raper et al 1996). The efficiency with which hepatocytes were harvested and transduced was low, 30% and 10% respectively (Grossman et al 1995). Protocols for lentiviral-mediated gene therapy have improved with in vitro transduction efficacy reaching 90% in non-human and human hepatocytes (Nguyen et al 2009). Hepatocyte transplantation, however, remains relatively inefficient and variable, likely due to poor engraftment, limited persistence of engrafted hepatocytes and the lack of a proliferative advantage (Gramignoli et al 2015).

Retroviral-mediated in vivo gene therapy was well tolerated when vector was administered intravenously in haemophilia A patients, but no significant clinical benefits were observed (Powell et al 2003). Preclinical studies failed to accurately predict the therapeutic dose and it was suggested that retroviral vectors were unable to transduce non-dividing hepatocytes (Chuah et al 2004). Therefore, alternative viral vectors, including lentiviral vectors, have been developed for treatment of hemophilias. Indeed two promising approaches with lentiviral vectors are in preclinical development: i) systemic lentiviral-mediated liver-restricted gene therapy in a dog model of haemophilia B, which showed long-term efficacy, induction of liver tolerogenic properties in stimulating CD4^+^CD25^+^FoxP3^+^ regulatory T cells and no evidence of genotoxicity in mice (Cantore et al 2015). The immune tolerance is of particular interest for patients with anti-FIX inhibitors, who are currently excluded from gene therapy trials; ii) ex vivo transduction of haematopoietic stem cells (HSC) in mice with successful *FIX* gene expression to target cells of the erythroid (Chang et al 2008) or the megakaryocyte lineage (Chen et al 2014). A lentiviral vector is currently in preclinical development for haemophilia B (Dolgin 2016). Lentiviral vectors are able to accommodate large transgenes such as *FVIII* gene for haemophilia A (Kuether et al 2012). Lentiviral-mediated liver-directed ex vivo gene therapy has been successfully reported in a pig model of tyrosinaemia 1 using the selective advantage of *Fah* ^*+/+*^ modified hepatocytes (Hickey et al 2016).

The risk of insertional mutagenesis has been reported with retroviral vectors (Cavazzana-Calvo et al 2000; Mukherjee and Thrasher 2013), however, there is evidence that the risk is lower with lentiviral vectors (Kotterman et al 2015). To improve safety, self-inactivating (SIN) vectors have been developed in which LTR enhancer/promoter elements in the U3 region have been deleted (Miyoshi et al 1998; Zufferey et al 1998). This, however, does not completely eliminate the risk of insertional mutagenesis as heterologous enhancer-promoter elements still need to be included in vector constructs. Genotoxicity has been observed after foetal injections of non-primate and primate SIN-lentiviral vectors (Nowrouzi et al 2013; Condiotti et al 2014). So far, more than 125 patients over 14 years have been treated with haematopoietic stem cells or T cells transduced by lentiviral vectors with no oncogenic event reported (Cartier et al 2009; Biffi et al 2013; McGarrity et al 2013; Booth et al 2016). Another approach relies on the mutation of the integrase protein to generate non-integrating or integration-deficient lentiviral vectors (Nightingale et al 2006; Philippe et al 2006; Yanez-Munoz et al 2006), which have shown long-lasting gene expression in non-dividing tissues (Apolonia et al 2007; Rahim et al 2009) and phenotype correction in mice with haemophilia B (Suwanmanee et al 2014).

## Adenoviral vectors

*Adenoviruses* are non-enveloped double-stranded DNA viruses with a large 36 kb genome and are capable of transducing dividing and non-dividing cells. In humans, a common target is epithelial cells of the respiratory or gastrointestinal tracts causing mild upper respiratory tract infection, gastroenteritis, or asymptomatic seroconversion. More than 55 serotypes are described but most vectors are derived from endemic serotypes 2 and 5 (Piccolo and Brunetti-Pierri 2014). The seroprevalence against the most common serotype (Adenovirus 5) is high with neutralising antibodies in 45–80% (Kotterman et al 2015). Adenoviruses elicit a sustained innate and cytotoxic-mediated immunity, which leads to the clearance of transduced cells (Tang et al 2006; Thaci et al 2011). Neither adenoviruses nor adenoviral vectors have been associated with genotoxicity in humans (Stephen et al 2010). Adenoviral vectors can accommodate large transgenes, which remain episomal in the transduced cell but enable a long-lasting transgene expression in quiescent or dividing cells. In animal models, adenoviral vectors exhibit strong liver tropism through interaction with coagulation factor X (Kalyuzhniy et al 2008; Vigant et al 2008; Waddington et al 2008).

### Clinical applications and limitations

In adenoviruses, expression of genes occurs in two early and late phases. The early phase is mediated by E1-E4 transcription units. Proteins encoded by E1 are essential for viral gene expression and DNA replication. Late gene expression is mediated by an internal promoter (Benihoud et al 1999). First-generation adenoviral vectors have E1 or E1-E3 regions removed and are theoretically replication-defective. However, these vectors keep a mild “leaky” expression of viral genes and are still able to synthesise some viral proteins, likely due to E1-like protein in the target cells (Zhang et al 1998; Lozier et al 1999). Moreover, the immunogenic properties of first-generation adenoviral vectors cause severe acute innate and chronic adaptive immune responses in small and large animal models (Yang et al 1994). To reduce this immune response, deletion of transcription units E1/E2/E3, E1/E4/E3, E1/E2/E3/E4) has been introduced (Alba et al 2005). Only the last combination achieved a reduction of vector toxicity, but with a reduced duration of the transgene expression (Gao et al 1996; Raper et al 1998; Andrews et al 2001). A second generation (E1- and E4- deleted) adenoviral 5 vector was used for the OTC deficiency trial, in which Jesse Gelsinger, a young adult with late-onset OTC deficiency enrolled in the highest dose group, died after developing a fatal acute toxic reaction with fulminant inflammatory response and multi-organ failure hours after the injection of the vector (Raper et al 2003). It has been hypothesised that this was caused by an innate immune response with a cytokine storm triggered by antigen presenting cells against capsid proteins (Raper et al 2003). The reason for severity of this immune response remains unclear as another patient injected with the same dose exhibited only mild flu-like symptoms. A genetic predisposition or an immune memory response caused by pre-exposure to adenoviruses might partly explain this discrepancy (Wilson 2009). In this trial, safety issues were surprisingly not dose-related and had not been predicted to this extent by animal studies (Raper et al 2002). Furthermore, no significant clinical benefit was observed (Raper et al 2002).

To limit this immune response, “gutless” or helper-dependent adenoviral vectors (HD-Ad) have been designed by deletion of all coding regions except the ITRs and the packaging signal (Ψ) required for the encapsidation of the adenoviral genome, which have been replaced by the transgene cassette and stuffer DNA (Alba et al 2005). This new generation of vectors have shown an improved safety profile, exhibiting a reduced acute innate immune response and an absence of chronic toxicity. In first-generation adenoviral vectors, the “leaky” expression of the remaining viral genes have a direct cytotoxic effect and triggers an adaptive cellular immune response directed against the transduced cell, which in turn results in transient transgene expression and chronic toxicity (Brunetti-Pierri and Ng 2011). As HD-Ad do not contain any viral genes, this late toxicity is not observed, which allows a long-term transgene expression as observed in small (Kim et al 2001; Toietta et al 2005) and large animal models (Morral et al 1999; Brunetti-Pierri et al 2006). This has allowed successful long-term phenotypic correction of various liver monogenic disorders (Brunetti-Pierri and Ng 2011), among which haemophilia A (Reddy et al 2002; Hu et al 2011), haemophilia B (Ehrhardt and Kay 2002), OTC deficiency (Mian and Lee 2002), glycogen storage disease 1A (Koeberl et al 2007), Criggler-Najjar syndrome (Schmitt et al 2014), primary hyperoxaluria type 1 (Castello et al 2016), acute intermittent porphyria (Unzu et al 2013), phenylketonuria (Cerreto et al 2012), familial hypercholesterolaemia caused by mutations in the *LDLR* (Nomura et al 2004) and *ApoE* genes (Belalcazar et al 2003) in rodents, haemophilia A (Brown et al 2004; McCormack et al 2006) and B (Ehrhardt et al 2003; Brunetti-Pierri et al 2005), glycogen storage disease type 1 (Crane et al 2012) in dogs, and haemophilia A and B and Pompe disease (Rastall et al 2016) in non human primates.

However, the acute innate immune response directed against capsid proteins is not abolished in HD-Ad vectors (Muruve et al 2004). For both first-generation and helper-dependent adenoviral vectors, this acute immune response is dose-dependent and can be lethal at high doses in non human primates (Morral et al 2002; Brunetti-Pierri et al 2004). Differences in the severity of the immune response between species have emerged due to variable interactions between blood cells and hepatic microarchitecture such as size of liver sinusoidal fenestration (Piccolo and Brunetti-Pierri 2014). The activation of this innate immunity is multifactorial. Adenoviral particles can trigger the immune response by binding to Toll-like receptors (TLR2, TLR9) at the surface of antigen presenting cells, and/or activate the complement cascade in the bloodstream (Kiang et al 2006; Zhu et al 2007). Kupffer cells recognise the adenoviral capsid either via antibody-mediated opsonisation or in binding complement factors. Kupffer cells develop a pro-inflammatory state with necrotic death, which further disseminate the immune response (Schiedner et al 2003).

In vivo HD-Ad mediated gene therapy has been performed in one patient in a phase I trial for haemophilia A. After a single intravenous low-dose injection, the patient developed flu-like symptoms with transient fever, chills, back pain, headache and transient biological abnormalities including thrombocytopenia, laboratory features of disseminated intravascular coagulopathy, increase in interleukin 6 levels, and elevated liver transaminase levels peaking at 7 days (marked as grade 3 liver toxicity) (Chuah et al 2004; White and Monahan 2005; Chandler and Venditti 2016). The patient expressed 1% FVIII for some months but the trial was halted for safety reason although biological abnormalities came back to normal within 19 days. Unfortunately, this trial has not been published in a peer-reviewed format and few details are available (Piccolo and Brunetti-Pierri 2014). The cause of these symptoms remains unclear although, as supposed for the OTC trial involving Jesse Gelsinger, the innate immune response against the adenoviral capsid and its subsequent release of cytokines has been suspected (Chuah et al 2004). Contamination by an adenoviral helper virus remains a possible explanation (Chuah et al 2004).

Despite limited application for liver monogenic disorders, adenoviral vectors have been successfully used for oncolytic virotherapy (Rosewell Shaw and Suzuki 2016) and vaccination (Majhen et al 2014), which exploits adenoviral immunogenicity.

## Adeno-associated viral vectors

**Fig. 2:**
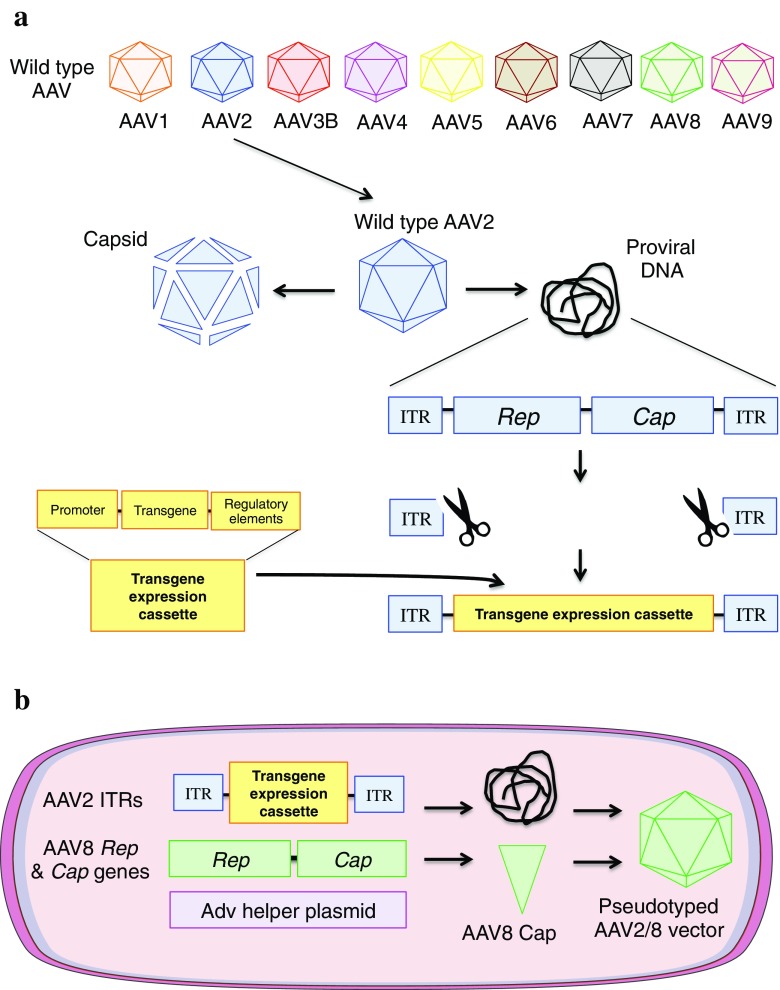
Synthesis of an AAV vector. **(a)** Initially, the single-stranded proviral DNA is excised to remove *Rep* and *Cap* genes from different wild type AAV serotypes. The transgene expression cassette containing the promoter, the transgene and various regulatory elements is cloned between the 2 ITRs, which are the only wild type AAV sequences retained. (**b**) For vector synthesis, triple transfection of three plasmids is performed in a packaging cell with proviral plasmid encoding the recombinant viral genome, a plasmid containing Rep and Cap and a helper plasmid. “Pseudotyped” AAV vectors contain ITRs from a specific AAV serotype (usually AAV2) and a *Cap* gene encoding viral proteins (VP1, 2 and 3) from a different serotype (*e.g* AAV8) in order to provide organ-specific transduction of the recombinant AAV vector named AAV2/8. AAV: adeno-associated virus; Adv: adenovirus; ITR: inverted terminal repeat

*Adeno-associated viruses* (AAV) are non-enveloped, single-stranded DNA viruses that belong to the *Dependovirus* genus and the *Parvoviridae* family. Initially identified as a contaminant of an adenoviral preparation (Atchison et al 1965), the virus was later shown to require co-infection with a helper virus to replicate. In the absence of helper virus, AAV can enter target cells and establish latent infection through genomic integration and/or formation of episomes. AAV is widely considered non-pathogenic and has yet to be definitively linked to disease causation. AAVs can transduce dividing and non-dividing cells. The seroprevalence against the most common serotype AAV2 is 40–60% (Louis Jeune et al 2013). AAV virions consist of an icosahedral capsid of approximately 22 nm in diameter enclosing a 4.7 kb single-stranded genome. The genome is flanked by two 145 nucleotide inverted terminal repeats (ITRs) containing all of the necessary *cis*-acting functions for proviral rescue, genome replication and packaging. The viral genome encodes 4 Rep proteins required for proviral rescue and genome replication, and three viral proteins VP1, VP2 and VP3, which assemble to form the capsid (Fig. 2A) (Samulski and Muzyczka 2014). Some, but not all, AAV capsid serotypes (Earley et al 2017) require expression of an assembly-activating protein (AAP) encoded by an alternative reading frame of the *Cap* gene and providing scaffolding activity (Naumer et al 2012). Numerous AAV serotypes have been isolated from humans, non-human primates and other species, with the viral capsid determining species and target cell tropism through interaction with a diversity of cell surface receptors/co-receptors and intracellular trafficking pathways that remain incompletely understood. A multi-serotype AAV receptor has been recently identified (Pillay et al 2016), but its precise role in uptake and trafficking has yet to be elucidated (Summerford and Samulski 2016). For example, AAV3B uses the human hepatocyte growth factor (hHGF) receptor, which restricts transduction to primates and especially to the liver (Vercauteren et al 2016).

Since 2004, *AAV vectors* have emerged as the leading candidates for gene therapy in monogenic liver disorders with the best accepted benefit-risk ratio (Dolgin 2016). Therefore, the following sections focus on this gene transfer approach detailing clinical successes and current limitations.

**Fig. 3:**
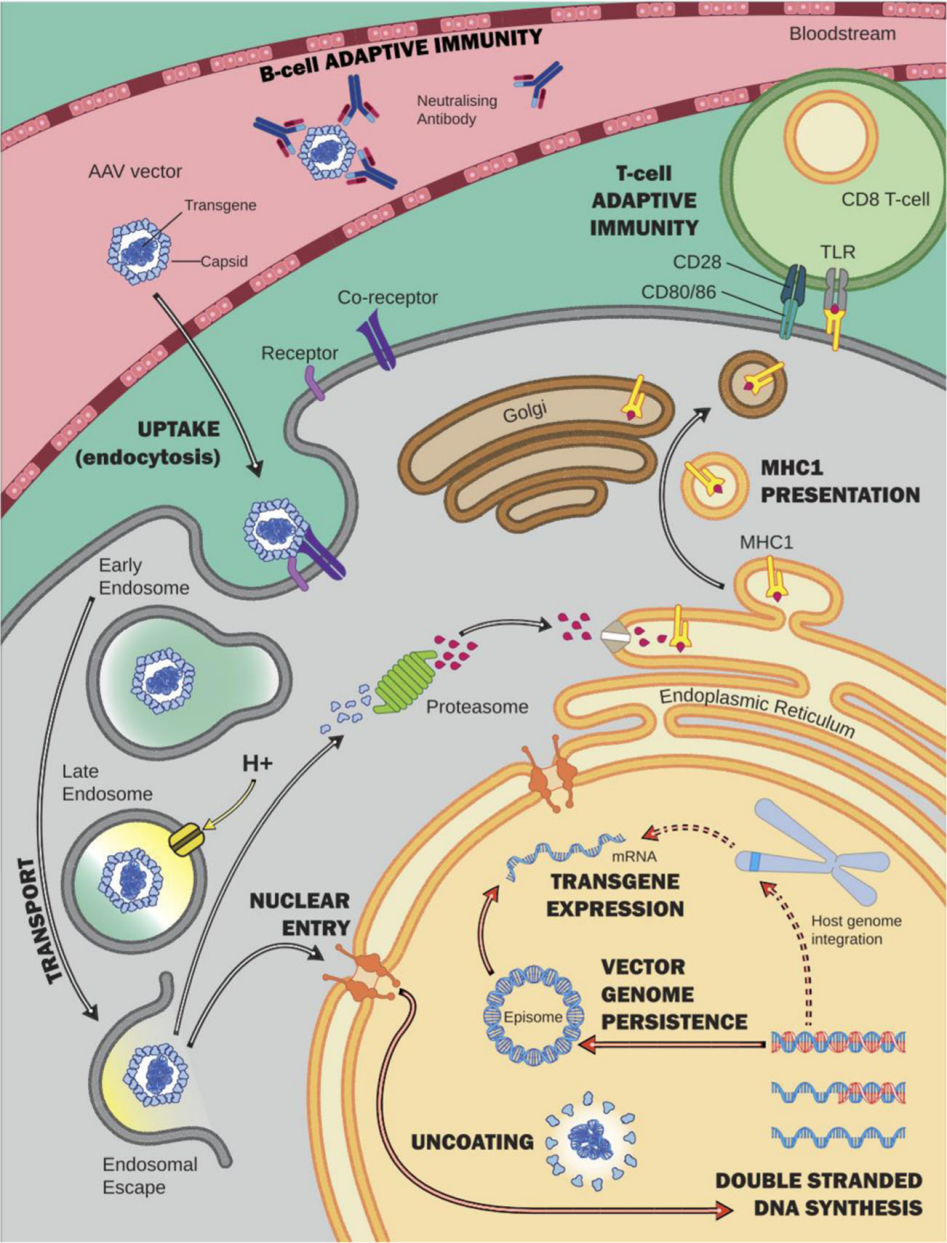
AAV vector uptake, in-cell processing and initiation of the immune response. Fenestrated endothelium of hepatic sinusoids allows the AAV vector to freely reach the hepatocyte. Once reaching the target cell, the vector binds an extracellular receptor and co-receptor specific to the capsid motifs. After an uptake by endocytosis, the vector is trafficked in the cytoplasm in early then late endosome. Acidification of the endosome modifies the capsid conformation. After endosomal escape, the AAV vector enters the nucleus via the nuclear pore complex. Capsid uncoating and release of the proviral DNA precede the synthesis of the 2nd strand of DNA. The viral genome then persists either as a non-integrated single- or double-stranded episome (99%) or (small percentage) integrates into the host genome (1%). Expression of the transgene is followed by synthesis of the protein of interest. Cell-mediated immune responses are initiated by the degradation of capsid or the transgene product (protein) in the proteasome and presentation at the surface of the transduced cell via the major histocompatibility complex I. CD8^+^ T cells recognise the antigen at the cell surface and initiate the immune cascade. Neutralising antibodies bind to the vector in the bloodstream and impair or prevent successful transduction of the organ target. MHC1: major histocompatibility complex I

AAV2 is the most widely studied serotype and was the first to be vectorized. To generate a recombinant AAV vector the native *Rep* and *Cap* genes are removed and replaced by a transgene expression cassette with only the flanking ITRs retained (Fig. 2A). Recombinant virus is produced by supplying *Rep* and *Cap* and necessary adenoviral helper functions in *trans*. A major development in AAV vector technology was the demonstration that recombinant AAV2 genomes can be cross-packaged, or pseudo-serotyped, with the capsids from other AAV serotypes (Rabinowitz et al 2002). This has dramatically broadened the cell types that can be efficiently targeted with AAV vectors. For example, pseudo-serotyping a recombinant AAV2 vector genome with the AAV8 capsid (designated AAV2/8) enhances tropism for hepatocytes, particularly in the mouse (Fig. 2B). AAV vectors bind to target cells via specific receptors and co-receptors that differ in a capsid-dependent manner and are taken up by endocytosis or macropinocytosis, before being trafficked to the nucleus for capsid uncoating. The uncoated genomes can remain in the nucleus in single-stranded form, be converted to double-stranded episomes or undergo genomic integration (Fig. 3) (Berry and Asokan 2016). Conversion of input single-stranded genomes to double-stranded transcriptionally active forms occurs with variable efficiency in different cell types. Self-complementary (*sc*) vectors differ from single-stranded vectors (*ss*) in that they contain a self-complementary transgene cassette that folds back on itself to form double-stranded DNA thereby bypassing the requirement for second strand synthesis, which is considered as a rate-limiting step for transgene expression. As a consequence the packaging size of the transgene cassette in *sc*AAV is reduced by half (McCarty 2008).

## Clinical successes of liver-directed AAV-mediated gene therapy

A rapidly increasing number of publications have reported proof-of-concept for AAV-based gene therapy in animal models for various inherited liver disorders including urea cycle defects, organic acidurias, phenylketonuria, glycogen storage disease type Ia, long chain fatty acid oxidation disorders, homozygous familial hypercholesterolemia, primary hyperoxaluria type I and progressive familial intrahepatic cholestasis (Hastie and Samulski 2015; Junge et al 2015).

In parallel, pioneering trials have been conducted since the 2000s, two of which targeted haemophilia B. This disease is an attractive target for gene therapy as an increase in plasma factor IX (FIX) of as little as 1% can confer significant phenotypic improvement. Haemophilia B is a burden in public healthcare systems with an annual cost of $300,000 for severely affected patients (Angelis et al 2015).

In 2004, a *ss*AAV2.ApoE/hAAT.*hFIX* vector, administered via the hepatic artery, showed a transient increase of plasma FIX from <1% to 3–11% over 4 weeks followed by a gradual decline over 4–8 weeks concomitant with transient asymptomatic rise in transaminases levels (Manno et al 2006), later recognised as T cell-mediated cytotoxicity (Mingozzi et al 2007).

In 2009, Nathwani et al, injected a *sc*AAV2/8.LP1.*hcoFIX* vector via a peripheral intravenous route and elicited a long-lasting (>5 years) increase of plasma FIX from <1% to 1–8% (Nienhuis et al 2016). Elevated transaminases occurring 7–10 weeks post-injection resolved after an oral course of corticosteroids, but were associated with a decrease of 50–70% in plasma FIX levels attributable to a cellular immune response against capsid epitopes (Nathwani et al 2014).

D’Avola et al, recently reported results of a trial of *sc*AAV2/5.hAAT.*hcoPBGD* vector in acute intermittent porphyria with peripheral intravenous delivery. No vector-related safety issues were reported and the rate of disease-related hospitalisation decreased, potentially as a consequence of closer metabolic follow-up. No change was observed in the levels of metabolic biomarkers (D’Avola et al 2016). This might be explained by a less efficient liver transduction mediated by AAV5 relative to AAV8 and a reduced expression of the episomal transgene compared to the endogenous gene of interest (Baruteau et al 2017). However, no liver biopsy data was available to address this assumption.

Preliminary reports from ongoing clinical trials have confirmed Nathwani’s promising results for haemophilia B. After a single intravenous injection of AAV vectors with different capsids encoding the FIX gene or its Padua FIX variant, which contains a gain-of-function mutation, reported stabilised plasma FIX levels have ranged from 3 to 8% in the AMT-060 trial sponsored by Uniqure (Miesbach et al 2016) and the DTX-101 sponsored by Dimension Therapeutics (http://dimensiontx.com) to 20–44% in the high-dose cohort of the BAX 335 trial sponsored by Shire (Monahan et al 2015) and in the SPK-9001 trial sponsored by Spark Therapeutics/Pfizer (George et al 2016). In a haemophilia A gene therapy trial, BioMarin reported plasma factor VIII (FVIII) from 4 to 60% in the high-dose group of the BMN 270 trial (Pasi et al 2016) (Table 1). Importantly, endogenous FVIII is primarily secreted by endothelial cells (Fahs et al 2014). All the AAV-based trials have so far involved only adult patients, who had an undetectable baseline titre of neutralising antibodies to the capsid (usually accepted cut-off of 1/5 serum dilution). Monogenic liver disorders in AAV-based gene therapy development pipelines of pharmaceutical companies include OTCD, glycogen storage disease type Ia, citrullinemia type I, phenylketonuria, Wilson disease, methylmalonic acideamia and Crigler-Najjar syndrome (Kattenhorn et al 2016).

**Table 1:** Clinical trials of gene therapy products for liver monogenic disorders. Intraportal and intrahepatic routes of administration relate to injection on the portal vein and the hepatic artery respectively. FVIII: factor VIII; FIX: factor IX; HD-adenovirus: helper-dependent adenovirus; LDL: low density lipoprotein; MoML: Moloney murine leukaemia virus; NA: not applicable; OTC: ornithine transcarbamylase; PBGD: porphobilinogen deaminase. Information sources: *clinicaltrial.gov* (accessed 06/01/2017) and company websites. If the date of the start of the trial was not available in *clinicaltrials.gov* website, the date of publication of the results is mentioned. The list of trials announced for 2017 is indicative and does not pretend to be exhaustive

Year (start of trial)	Viral vector	Disease	Product	Therapeutic	Sponsor	Route	Dose	Number of treated patients	Status	Reference	Clinicaltrial.gov identifier
1992	MoMLV Retrovirus 5	Homozygous familial hypercholesterolaemia	NA	LDL receptor gene	University of Michigan Ann Arbour	Intraportal (Ex vivo approach)	1 to 3.3 × 10e9 hepatocytes	5	Terminated	Grossman et al 1994 Grossman et al 1995 Raper et al 1996	NCT00004809
1998	Adenovirus 5	Ornithine transcarbamylase deficiency	NA	OTC gene	University of Pennsylvania	Intrahepatic	2 × 10e9 to 6 × 10e11vg/kg	18	Terminated	Raper et al 2003	NCT00004498
1999	AAV2	Haemophilia B	NA	Factor IX gene	NA	Intramuscular	2 × 10e11 to 1.8 × 10e12vg/kg	8	Terminated	Kay et al 2000 Manno et al 2003	NA
2003	MoMLV Retrovirus	Haemophilia A	NA	FVIII gene	Chiron	Intravenous	2.8 × 10e7 to 4.4 × 10e8vg/kg	13	Terminated	Powell et al 2003	NA
2004	HD-Adenovirus	Haemophilia A	NA	FVIII gene	GenStar	Intravenous	4.3 × 10e10vg/kg	1	Terminated	Chuah et al 2004	NA
2004	AAV2	α1-antitrypsin	NA	hAAt	University Massachussets	Intramuscular	2.1 × 10e12 to 6.9 × 10e13vg	12	Terminated	Flotte et al 2004 Brantly et al 2006	NCT00377416
2004	AAV2	Haemophilia B	NA	Factor IX gene	Avigen	Intrahepatic	8 × 10e10 to 2 × 10e12vg/kg	7	Terminated	Manno et al 2006	NCT00076557
2006	AAV1	α1-antitrypsin	NA	hAAT	University Massachussets	Intramuscular	6.9 × 10e12 to 6 × 10e13vg	9	Terminated	Brantly et al 2009	NCT00430768
2009	AAV2/8	Haemophilia B	NA	Factor IX gene	St Jude Children’s Hospital	Intravenous	2 × 10e11 to 2 × 10e12vg/kg	10	Recruiting	Nathwani et al 2014	NCT00979238
2010	AAV1	α1-antitrypsin	NA	hAAT	Applied Genetic Technologies	Intramuscular	6 × 10e12 to 6 × 10e12vg/kg	9	Terminated	Flotte et al 2011	NCT01054339
2012	AAV2/8	Haemophilia B	BAX 335	Padua mutant factor IX gene	Shire	Intravenous	2 × 10e11 to 3 × 10e12vg/kg	6	Terminated	Monahan et al 2015	NCT01687608
2014	AAV2/5	Acute intermittent porphyria	NA	PBGD gene	Digna Biotech	Intravenous	5 × 10e11 to 1.8 × 10e13vg/kg	8	Terminated	D’Avola et al 2016	NCT02082860
2015	Engineered AAV	Haemophilia B	SPK-9001	Padua mutant factor IX gene	Spark Therapeutics	Intravenous	5 × 10e11 vg/kg (Low dose)	7	Recruiting	George et al 2016	NCT02484092
	AAV2/5	Haemophilia B	AMT-060	Factor IX gene	Uniqure Biopharma	Intravenous	5 × 10e12 to 2 × 10e13vg/kg	5	Recruiting	Miesbach et al 2016	NCT02396342
	AAV2/rh10	Haemophilia B	DTX101	Factor IX gene	Dimension Therapeutics	Intravenous	1.6 × 10e12 to 1 × 10e13vg/kg	Undisclosed	Recruiting	clinicaltrials.gov	NCT02618915
	AAV2/5	Haemophilia A	BMN 270	Factor VIII gene	BioMarin Pharmaceuticals	Intravenous	6 × 10e12 to 6 × 10e13vg/kg	9	Recruiting	Pasi et al 2016	NCT02576795
2016	AAV2/6	Haemophilia B	SB-FIX	Factor IX integrating in the albumin locus via Zinc-finger-nuclease	Sangamo Bioscines	Intravenous	cDNA 4 × 10e12 to 4 × 10e13vg/kg and ZFN 5 × 10e11 to 5 × 10e12vg/kg	Undisclosed	Recruiting	clinicaltrials.gov	NCT02695160
	AAV2/8	Homozygous familial hypercholesterolaemia	RGX-501	LDL receptor gene	University of Pennsylvania/Regenxbio	Intravenous	2.5 × 10e12 to 7.5 × 10e12vg/kg	Undisclosed	Recruiting	clinicaltrials.gov	NCT02651675
Announced for 2017	AAV2/8	Ornithine transcarbamylase deficiency	DTX301	OTC gene	Dimension Therapeutics	Intravenous	2 × 10e12 to 2 × 10e13vg/kg	Undisclosed	Recruiting	clinicaltrials.gov	NCT02991144
	Engineered AAV	Haemophilia A	SPK-8011	Factor VIII gene	Spark Therapeutics	Intravenous	Undisclosed	Undisclosed	Recruiting	clinicaltrials.gov	NCT03003533
	AAV2/8	Haemophilia A	GO-8	Factor VIII gene	University College London	Intravenous	6 × 10e11 to 6 × 10e12vg/kg	NA	Not yet recruiting	clinicaltrials.gov	NCT03001830
	Engineered AAV	Haemophilia B	FLT-180	Undisclosed	Freeline Therapeutics						
	AAV2/8	Haemophilia A	BAX-888	Factor VIII gene	Shire						
	AVV	Haemophilia A	DTX201	Factor VIII gene	Dimension Therapeutics/Bayer						
	AAV2/6	Haemophilia A	SB-525	Factor VIII integrating in the albumin locus via Zinc-finger-nuclease	Sangamo Biosciences						
	AAV2/8	Mucopolysaccharidosis VI	MeuSIX	ARSB gene	NA						
	AAV2/8	Crigler Najjar	AT342	UGT1A1 gene	Audentes Therapeutics						

## Current challenges

### Insertional mutagenesis

Despite more than 170 AAV-based human trials approved, ongoing or completed (http://www.abedia.com/wiley/(accessed 2017 Jan 06)), no tumorigenic events have been reported so far. AAV vector genome mainly persists as episome in the transduced cell with a relatively low proportion of vector genomes undergoing integration preferentially in transcriptionally active genes, damaged DNA or enriched CpG islands (McCarty et al 2004). Experiments in neonatal mice have identified an increased risk of hepatocellular carcinoma (HCC) after systemic injection. This risk increased with the enhancer/promoter activity, younger age at time of injection and vector dose (Donsante et al 2007; Chandler et al 2015; Chandler et al 2017). Analysis of integration sites identified a rodent-specific hotspot in the *Rian* locus. Integration studies from human trials have not shown such hotspots, but rather a genome wide integration pattern involving neither HCC-related genes nor the human *Rian* homologue, *Dlk1-Dio3* (Kaeppel et al 2013; Gil-Farina and Schmidt 2016).

Controversies remain regarding the possible insertional mutagenic effects of wild type AAV. Detection of a clonal expansion of wild type AAV2 sequences in 11/193 HCCs within HCC-related genes (Nault et al 2015) initiated a passionate and unresolved debate about “driver” or “passenger” cancer-related genetic modifications (Berns et al 2015; Buning and Schmidt 2015). The cumulative safety experience with the rapidly growing number of AAV-based trials targeting the human liver, combined with the low rate of HCC-associated AAV integrations despite the high seroprevalence of wild type AAV in the human population (e.g. >50% for AAV2) (Thwaite et al 2015) are consistent with a favourable safety profile of AAV vectors. Nevertheless the findings of Nault et al warrant further studies and mandate close monitoring in ongoing human trials.

### Immune response

After vector delivery, non-specific innate immunity triggers both type I interferon signalling involved in transgene silencing (Suzuki et al 2013) and the release of proinflammatory cytokines (Jayandharan et al 2011). Highly-specific and long-lasting adaptive immunity generates B- and T-cell responses against the capsid and/or the transgene product (Fig. 3). Neutralising antibodies against the capsid, even at low titers, inhibit transduction after systemic delivery (Jiang et al, 2006a, b). This barrier is of substantial concern to gene therapy development and the ongoing liver-directed trials are recruiting only seronegative patients without neutralising antibodies against the AAV capsid. This narrows the target population as the seroprevalence against liver-specific AAV serotypes ranges from 20 to 30% for AAV5, 6 and 8 to 50–60% for AAV2 (Louis Jeune et al 2013). Cross-reactivity between serotypes is commonly >50% (Boutin et al 2010). This seroprevalence varies depending on geographic origin (Calcedo et al 2009) and age. Neonates receive maternal antibodies by transplacental transfer and acquired with maternal milk, which are lost over the first months of life. Thereafter, seroprevalence remains negligible until 3 years of age after which the seroconversion rate progressively increases until adulthood (Calcedo et al 2011; Li et al 2012).

Human CD8^+^ T-cell mediated immune responses are involved in AAV hepatotoxicity and were initially encountered during the first haemophilia B trial (Manno et al 2006). Capsid epitopes, presented via the major histocompatibility complex I (MHC1), were shown to drive expansion of a pre-existing pool of CD8^+^ memory T cells acquired during a previous co-infection of wild type AAV and helper virus (adenovirus or herpes virus for example). This response was dose-dependent (Mingozzi and High 2013) and could be stimulated by alternate capsids (Mingozzi et al 2007).

Hepatic tolerogenic properties involve the proliferation of a specific T cell subset, CD4^+^CD25^+^FoxP3^+^ Treg cells (Cooper et al 2009) interacting with Kupffer cells (Breous et al 2009). Expansion of these cells suppresses cytotoxic immunity against AAV-transduced hepatocytes and induces immunotolerance, e.g. after neonatal injection (Shi et al 2013). Regulatory tolerance requires continuous antigen presentation and has been successfully induced with transgenic proteins (Shi et al 2013; Perrin et al 2016). In contrast, capsid proteins are rapidly eliminated in the proteasome (Berry and Asokan 2016) and therefore very unlikely to induce tolerance.

Various approaches aim to overcome these unwanted immune responses in order either to treat seropositive patients or to prevent sensitisation against the AAV capsid, which would allow reinjection in the future. Capsid modification targeting specific epitopes can evade host immunity (Tseng and Agbandje-McKenna 2014). Any strategy optimising the transduction of the target organ such as optimised expression cassette design, or capsid modifications will in turn decrease the amount of vector required for a similar effect. The decrease in vector dose will further reduce the immune response (Mingozzi and High 2011). Various protocols involving transient immunosuppression have been proposed in large animal models and humans. These include plasmapheresis (Monteilhet et al 2011; Chicoine et al 2014), monoclonal antiCD20 antibody (rituximab) (Mingozzi et al 2013; Corti et al 2014), non-depleting antiCD4 antibody (McIntosh et al 2012), sirolimus (Corti et al 2014), cyclosporine A (McIntosh et al 2012; Mingozzi et al 2012), tacrolimus with mycophenolate mofetil (Chicoine et al 2014), proteasome inhibitors, e.g. bortezomib (Monahan et al 2010) and corticosteroids (Flanigan et al 2013; Chicoine et al 2014; Nathwani et al 2014). One currently controversial strategy to blunt anti-capsid immune responses, is to co-inject “full” AAV vectors and “empty” capsids as decoys, in an attempt to competitively bind existing antibodies (Tse et al 2015).

### Optimised targeting

The ideal AAV vector would exclusively transduce the desired target cells. This would limit unwanted immune responses, avoid ectopic transgene expression and further reduce the already low risk of germline transmission. Extensive capsid-focused research based on high-throughput in vitro and in vivo screening of “libraries” of new capsid variants is ongoing in order to optimise vectors for specific applications. These “accelerated evolution” libraries are generated using strategies such as error-prone PCR (Kotterman and Schaffer 2014; Deverman et al 2016) and “capsid shuffling” with random cut-paste sequences of wild type *cap* genes (Kay 2011; Louis Jeune et al 2013; Choudhury et al 2016). This approach is paying off by generating re-engineered AAV variants with increased transduction efficiency in primary human hepatocytes (Lisowski et al 2014).

### Germline transmission

The risk for this phenomenon is difficult to quantify. Insertion of AAV sequences into the genome of a gamete could potentially interfere with normal foetal development or promote tumorigenicity in the progeny. So far adult patients enrolled in AAV trials with systemic delivery have been required to use contraception. Vector sequences have been detected transiently in semen of treated patients in AAV2 (Manno et al 2006) or AAV8 (Nathwani et al 2014) trials with the latest clearance of the vector observed at 12 weeks post-injection, quicker in younger men, but not in an AAV5 trial (D’Avola et al 2016). Vector was observed in seminal fluid but not in motile sperm and spermatogonia aligned with previous studies (Arruda et al 2001; Couto et al 2004).

### Suboptimal animal models

**Fig. 4:**
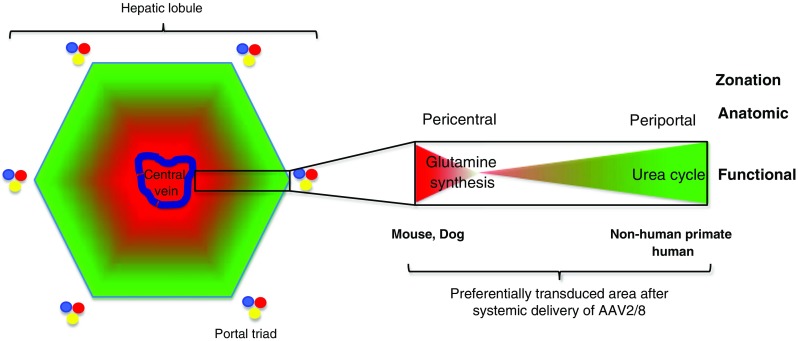
Species-related differences in transduction of the hepatic lobule by AAV vector compared with metabolic zonation for ammonia clearance: example with AAV2/8 vector

The following examples demonstrate why animal experiments provide limited value for predicting effects in human trials:

- T-cell mediated cytotoxicity observed in the first haemophilia B trial was not predicted by experiments in mice, dogs or non-human primates (Pien et al 2009). Unlike research animals, humans are exposed to wild type AAV infections generating anti-AAV memory T cells, which are reactivated at the time of vector exposure (Mingozzi et al 2007).
- An over 80% rate of HCC was observed in a mouse model of methylmalonic acidaemia injected neonatally with AAV2/8. Integration occurred in the *Rian* hotspot, a rodent-specific locus absent in other vertebrate genomes (Chandler et al 2015).
- The patterns of liver transgene expression in the hepatic lobule varies among different species. For example, using the AAV8 capsid, transgene expression is predominantly pericentral in mice and dogs and periportal in non-human primates (Fig. 4) (Bell et al 2011). This is of particular importance for liver diseases where metabolic zonation underpins that certain metabolic functions occur predominantly in certain areas of the liver lobule, which is the functional unit of the liver. For example, the urea cycle activity mostly takes place in periportal hepatocytes (Gebhardt and Matz-Soja 2014). To achieve adequate control of severe hyperammonaemia in the OTCD mouse model, therefore, requires a much higher than expected dose of AAV2/8 vector carrying OTC transgene, which might be explained by the non-physiological pattern of liver transduction (Cunningham et al 2011). Thus, it is difficult to reliably extrapolate vector doses for human translation from studies in mice in liver diseases with metabolic zonation like OTCD.
- AAV2/8 vectors are capable of transducing 100% hepatocytes in adult mice (Cunningham et al 2008), but data from human trials in haemophilia B have shown an increase in plasma FIX only 2 to 8% (Nienhuis et al 2016), suggesting much less efficient AAV2/8-mediated hepatocyte transduction in humans. Interestingly, most studies in a chimeric FRG (*Fah* ^*−/−*^ */Rag2* ^*−/−*^ */Il2rg* ^*−/−*^) mouse-human liver model (Lisowski et al 2014; Wang et al 2015; Vercauteren et al 2016) showed that AAV3B and AAV3B-derived vectors (AAV3-ST, AAV-LK03) are able to transduce human hepatocytes approximately 10 times more efficiently than AAV2/8 whilst transduction of murine hepatocytes is minimal (Lisowski et al 2014).

The FRG mouse has a combination of tyrosinaemia type I and immunodeficiency phenotypes and is an attractive model to study human hepatocytes in vivo with the intention of overcoming limitations due to species-specificity (Azuma et al 2007). Human *Fah* ^*+/+*^ hepatocytes have a selective growth advantage relative to the *Fah*-deficient native mouse hepatocytes allowing human cell engraftment up to 90% of the liver mass (Azuma et al 2007). Moreover, this model can address disease-specific questions if engrafted with hepatocytes from patients with liver specific disorders. Recently, the even more complex FRGN (*Fah* ^*−/−*^ */Rag2* ^*−/−*^ */Il2rg* ^*−/−*^ */NOD*) mouse model has been described in which FRG mice, developed in a non-obese diabetic (NOD) mouse strain, are simultaneously co-transplanted with human hepatocytes and human haematopoietic stem cells (Wilson et al 2014).

### Limited capacity of AAV vectors

The single-stranded AAV vector can accommodate a transgene cassette of approximately 4.6 to 5 kb (Hirsch et al 2016). This capacity is reduced by half (2.3 kb) in self-complementary (i.e double-stranded) vectors. This is a major limitation compared to non-viral or other common viral vectors like lentiviral/retroviral vectors (up to at least 14 kb (Counsell et al 2017)) or helper-dependent adenoviruses (up to at least 38.9 kb (Suzuki et al 2011)). To deliver oversized transgenes, several approaches have been developed. Designing mini-promoters or mini-genes of interest can be successful (Yan et al 2015). Alternatively, dual AAV co-transduction has been successfully tested either with split AAV or fragment AAV (Hirsch et al 2016). Split AAVs use the inherent tendency for intermolecular genome association observed with AAV genomes via either homologous recombination (HR) or non-homologous end joining (NHEJ) to produce concatemers. The overlapping approach uses vectors A and B, which display a homology sequence to promote intermolecular homologous recombination (Duan et al 2001; Koo et al 2014). In the trans-splicing approach, two splice sites in 3′ cDNA of vector A and 5′ cDNA of vector B are recognised in concatemerized provirus to generate the single DNA molecule of the oversized gene of interest (Duan et al 2001). A combination of the two approaches is known as the hybrid trans-splicing technique (Trapani et al 2014). In fragment AAV, the transgene is not entirely encapsidated but only fragments of different size, which can recombine on overlapping regions (Hirsch et al 2016). A limitation common to these approaches is reduced functional transduction efficiency.

### Limited manufacturing capability

For the last couple of years, the rise in demand for good-manufacturing practice (GMP) AAV vectors for preclinical and clinical studies has created a bottleneck, delaying a number of projects. The industry is progressively taking up the gauntlet and developing improved and innovative methods for vector production in larger bioreactors with optimised reagents, purification techniques and packaging cell lines (Clement and Grieger 2016; Grieger et al 2016).

### Gene therapy requires an innovative economic model for success in modern healthcare

Patients with inherited metabolic disorders individually impose a much heavier financial burden on the healthcare system compared to the average person. For example, the lifetime cost for methylmalonic/propionic acidaemias and Gaucher disease is $1.5 and 5 million, respectively (Li et al 2015; Orkin and Reilly 2016). Gene therapy has the potential to achieve substantial savings. For example, a haemophilia B trial has shown that single injection of the gene therapy product in a cohort of ten patients can save more than $2.5 million over three years for the healthcare system in the UK (Nathwani et al 2014).

It is likely that to recover the investment in product development, companies will need pricing gene therapy treatments ambitiously when their products reach market. However, the cost of treatment will need to be affordable for public healthcare systems. The first gene therapy product approved by the European Medicines Agency (EMA), Glybera®, is marketed by Uniqure. This drug has been in development for 8 years, with the initial developer going bankrupt and a currently proposed market cost of $1.2 million per patient (Bryant et al 2013). GlaxoSmithKline‘s Strimvelis® is the second gene therapy product to reach the market and was approved by the EMA in 2016. This gene therapy product, which targets ADA-SCID has been in development for over 16 years and the proposed cost is $665,000 per patient (Hoggatt 2016; Schimmer and Breazzano 2016).

Over the last 20 years, greater than $4.3 billion have been spent on development of gene therapy technology and return on this investment is still awaited (Ledley et al 2014). Although most of the gene therapy programmes remain in the early stages of development, healthcare economists are generating models to cost treatments, which might provide lifelong cures. A pay-for-performance system has been proposed with yearly-capped annuity paid to the pharmaceutical company if criteria of a metabolic control of the disease are met (Touchot and Flume 2015). These criteria might reflect cost-effectiveness and not only cost-saving. This approach values the gain in quality of life estimated by quality-adjusted life years (QALY) analysis, which includes many parameters such as lifespan and ability to work (Orkin and Reilly 2016). Vouchers systems with longer financial incentives might be another option (Schimmer and Breazzano 2016).

In parallel, the regulatory framework is evolving with the progress of technology and the increasing experience being gathered from human trials. The Food and Drug Administration (FDA) and the EMA have published recommendations for gene therapy products (Narayanan et al 2014). The need for shorter and less expensive paths to clinical trials and conditional approval relying more on human data for safety and efficacy has now been recognised, as exemplified by the FDA’s Breakthrough Therapies programme and the EMA’s Adaptive Pathways and Priority Medicines (PRIME) schemes which were launched in 2012, 2014 and 2016, respectively (Mullard 2016). These more flexible pathways will need to be agreed by government funding bodies (Macaulay 2015).

## Considerations for paediatric application

Paediatric administration of gene therapy has several theoretical advantages (McKay et al 2011). These include prevention of early death or irreversible neurological sequellae, transduction of stem/progenitor cells and possible avoidance of immune response as neonates have an immature immune system, while infants and children have lower rates of pre-existing anti-AAV immunity. The potential for even earlier gene therapy intervention has been explored in late gestation foetal macaques by intrahepatic injection of *sc*AAV2/8 and *sc*AAV2/5 vectors. Plasma human FIX levels of 8–112% were observed during a median follow-up period of 14 months without evidence hotspot integration and HCC (Mattar et al 2011).

There is a theoretical risk, however, of increased tumorigenicity in the developing liver as observed in experiments performed in neonatal mice (Donsante et al 2007; Chandler et al 2015). A further challenge in the paediatric liver is the likely progressive loss of vector genomes over time in concert with hepatocellular proliferation. More than 90% of the AAV-delivered transgene cassettes exists as non-integrated episomes (Cunningham et al 2008). The human liver weight doubles at 4 months, 16 months, 6 years and 12 years of age, which means that the adult liver is 16 times heavier than the neonatal liver (Coppoletta and Wolbach 1933; Sunderman and Boerner 1949). Therefore, it is unlikely that a neonatal injection will be sufficient to provide lifelong correction of the phenotype in metabolic liver diseases, with reinjection during the phase of rapid liver growth likely to be necessary.

An alternative approach to reinjection could be the use of integrating vectors and or locus-specific genome engineering. Sangamo Therapeutics Inc. is developing tools for transgene integration into the albumin locus and uses zinc finger nucleases coupled with AAV technology. Other genome editing tools have been successfully tested with AAV vectors in a neonatal OTC deficiency mouse model. In these experiments, two AAV vectors were injected simultaneously, one of which encoded the transgene and the other the enzymatic system for integration or site specific cutting by Piggybac transposase (Cunningham et al 2015) and CRISPR-Cas9 (Yang et al 2016), respectively.

### Muscle-directed gene therapy for liver monogenic disorders

Muscle-directed gene therapy has been developed for liver monogenic disorders with secreted protein such as haemophilia A and B and α1-antitrypsin deficiency. Intramuscular injections might circumvent some caveats observed with systemic injection, the common route of delivery for liver-targeted gene therapy: limited biodistribution with reduced risk of germline transmission, minimal exposure to circulating neutralising antibodies, reduced dose of vector for a similar effect.

A proof of concept using AAV2 vectors in mice (Herzog et al 1997) and dogs (Herzog et al 1999) affected by haemophilia B paved the way for a clinical trial (Kay et al 2000; Manno et al 2003), which showed a safe profile with long-standing expression in some patients (Jiang et al, 2006a, b; Buchlis et al 2012) but only a mild increase in plasma factor IX around 1% (Manno et al 2003). Depending on the dose considered, dozens to hundreds of intramuscular injections are necessary, which makes this route particularly impractical. For instance, Manno et al administered between 10 to 90 injections per patients in lower limbs (Manno et al 2003). Similarly, a proof of concept in mice with haemophilia A has been reported (Mah et al 2003).

Three AAV-mediated clinical trials have been conducted for α1-antitrypsin deficiency. In this disorder, a plasma level of wild-type (M) α1-antitrypsin above 11 μM is considered reducing the risk of developing emphysema. A first trial based on an AAV2 capsid showed an acceptable safety profile with mild local reactions at the site of intramuscular injection (redness, tenderness, bruising) and a seroconversion against AAV2. Unfortunately, only one out of 12 patients demonstrated a minimal increase of plasma M α1-antitrypsin at 82 nM (Flotte et al 2004; Brantly et al 2006). Two other trials (phase I then phase II) were conducted with a vector based on AAV1 capsid known for its better muscle transduction compared to AAV2 (Flotte et al 2011). In both trials, minor side effects and a seroconversion against AAV1 were observed (Brantly et al 2009; Flotte et al 2011). A moderate infiltration of reactive T lymphocytes in muscle biopsies was noticed (Flotte et al 2011). Plasma levels of M α1-antitrypsin were still mild, although improving in the latest trial due to higher doses injected (412 to 694 nM in the highest dose group) but far from the targeted protective level of 11 μM (Flotte et al 2011).

## Conclusion

Over the last decade, major discoveries in the understanding of viral vector biology have generated promising results in pioneering clinical trials for haemophilia B using AAV vectors. This has paved the way for a wider development of AAV-mediated gene therapy for monogenic liver disorders. Although several clinical, manufacturing and economic challenges remain, this approach to treatment for severely debilitating diseases generated widespread enthusiasm shared by clinicians, researchers and investors alike. Gene transfer technologies are reaching an exciting threshold of efficacy and promise to revolutionise the management of many currently untreatable diseases.
